# Evaluating the Implementation and Feasibility of a Web-Based Tool to Support Timely Identification and Care for the Frail Population in Primary Healthcare Settings

**DOI:** 10.15171/ijhpm.2017.32

**Published:** 2017-03-07

**Authors:** Beverley Lawson, Tara Sampalli, Stephanie Wood, Grace Warner, Paige Moorhouse, Rick Gibson, Laurie Mallery, Fred Burge, Lisa G. Bedford

**Affiliations:** ^1^Building Research for Integrated Primary Healthcare (BRIC NS), Nova Scotia Primary & Integrated Health Care Innovations Network, Halifax, NS, Canada.; ^2^Primary Care Research Group, Dalhousie Family Medicine, Halifax, NS, Canada.; ^3^Dalhousie University, Halifax, NS, Canada.; ^4^Primary Health Care, Nova Scotia Health Authority, Halifax, NS, Canada.; ^5^School of Occupational Therapy, Dalhousie University, Halifax, NS, Canada.; ^6^Continuing Care, Nova Scotia Health Authority, Halifax, NS, Canada.; ^7^Healthy Populations Institute, Halifax, NS, Canada.; ^8^Palliative and Therapeutic Harmonization (PATH) Program, Halifax, NS, Canada.; ^9^Department of Family Practice, Nova Scotia Health Authority, Halifax, NS, Canada.

**Keywords:** Frail Elderly, Primary Healthcare (PHC), Patient Care Planning, Web-Based Portal

## Abstract

**Background:** Understanding and addressing the needs of frail persons is an emerging health priority for Nova Scotia and internationally. Primary healthcare (PHC) providers regularly encounter frail persons in their daily clinical work. However, routine identification and measurement of frailty is not standard practice and, in general, there is a lack of awareness about how to identify and respond to frailty. A web-based tool called the Frailty Portal was developed to aid in identifying, screening, and providing care for frail patients in PHC settings. In this study, we will assess the implementation feasibility and impact of the Frailty Portal to: (1) support increased awareness of frailty among providers and patients, (2) identify the degree of frailty within individual patients, and (3) develop and deliver actions to respond to frailtyl in community PHC practice.

**Methods:** This study will be approached using a convergent mixed method design where quantitative and qualitative data are collected concurrently, in this case, over a 9-month period, analyzed separately, and then merged to summarize, interpret and produce a more comprehensive understanding of the initiative’s feasibility and scalability. Methods will be informed by the ‘Implementing the *Frailty Portal in Community Primary Care Practice’* logic model and questions will be guided by domains and constructs from an implementation science framework, the Consolidated Framework for Implementation Research (CFIR).

**Discussion:** The ‘Frailty Portal’ aims to improve access to, and coordination of, primary care services for persons experiencing frailty. It also aims to increase primary care providers’ ability to care for patients in the context of their frailty. Our goal is to help optimize care in the community by helping community providers gain the knowledge they may lack about frailty both in general and in their practice, support improved identification of frailty with the use of screening tools, offer evidence based severity-specific care goals and connect providers with local available community supports.

## Background

Frailty is a distinct and recognizable health state in which multiple body systems gradually lose their built in reserve.^1,2^ While often associated with age, not all older adults are frail, and not all persons who are frail are older adults. What is known is that around 10% of people over the age of 65 experience frailty; this number increases to between 25%-50% for those over age 85.^2^ This syndrome is characterized by decreased physiologic reserve and susceptibility to health stressors, resulting from cumulative health deficits across multiple organ systems, and causing vulnerability to adverse health outcomes.^1^ It is generally associated with multi-system (eg, mobility, cognition, function, endurance) deterioration, and typically impacts the geriatric population. Persons experiencing frailty are highly susceptible to adverse events such as falls, hospitalization, disability, dependence, placement in long-term care and death.^2-4^ Simply put, each time a health challenge occurs (eg, an infection), the body’s ability to “bounce back” from health challenge is decreased. Not only is recovery time increased, but deterioration in physical and cognitive health, after what many would consider a minor health event, is common. Since frailty is a robust marker of vulnerability, appropriate care planning and care delivery requires the recognition of frailty when it is present.

Frailty can be better managed with early screening and intervention.^5^ To enable this screening and intervention to occur effectively and consistently, primary care providers need simple, validated, and effective tools.^6^ Recent advances in technology have enabled easy, timely and relevant access and application of required tools and standards at the point of care; yet challenges still exist with implementation into practice.^7^

The Nova Scotia Health Authority (NSHA) is the largest provider of health services in Nova Scotia, Canada, delivering healthcare and support services through hospitals, health centres, and community-based programs.^8^ Given that the majority of frail persons live in the community, strengthening primary healthcare (PHC) to assess and treat frail adults is crucial in supporting them to age in their preferred setting, gain timely access to appropriate community resources, and be supported through to end-of-life.^8-15^ In 2012, Primary Health Care and the Department of Family Practice, NSHA^[1]^, began development of a Frailty Strategy outlining 6 areas of focus – understanding; engagement; care; evaluation, research and knowledge implementation; information technology and management; and governance. Together these areas of focus aim to align new and existing frailty-focused initiatives across all organizational, community and societal sectors in the health region.

To address the above identified gaps and to improve early identification and intervention processes in primary care, the Frailty Portal, a novel technology-based solution, was developed as a first step to improve the application of relevant standards at the point of care.^9-14^ It sets the stage for aligning patient and family caregiver understanding of overall health needs and informed decision-making regarding preventive strategies,^8,16^ medical interventions,^17^ and surgical interventions.

## Objectives

In this study we assessed the implementation feasibility and impact of the Frailty Portal, in community primary care practice for the identification of, and response to frailty. Guided by the domains identified in the Consolidated Framework for Implementation Research (CFIR)^18^ we propose to: (1) Identify and understand factors influencing the implementation feasibility of the ‘Frailty Portal’ among frail patients, their caregivers and PHC providers, (2) Assess the impact of the ‘Frailty Portal’ on frail patients, their caregivers and PHC providers, and (3) Identify the core components required to successfully scale the initiative to a broader community of PHC providers within and across jurisdictions.

## Methods

### Methodology

#### Study Settings

Primary care practices in Halifax, Eastern Shore and West Hants, Nova Scotia, Canada.

#### Study Population

A convenience sample of approximately 15 primary care physicians and nurse practitioners (NPs) working in the management zone known locally as Central Zone will be recruited to participate in the study. Within this area, a range of practices in urban, suburban and rural environments are represented with patients of varying levels of health, health literacy and socio-economic resources. Physicians and NPs will use the Frailty Portal to screen, identify and develop appropriate care plans. Patients who have been screened by the participating physicians and NPs and/or their caregivers will also be recruited to participate in the study. We expect to recruit at least 15 patients and or their caregiver per participating physician/NP. Patients/caregivers will offer feedback around satisfaction and experience of care through surveys and interviews. Approximately 15 members from key stakeholder groups will be recruited to participate in interviews. Key stakeholder participants will include local and provincial healthcare decision-makers, managers, PHC providers and information technology specialists.

#### Intervention

The Frailty Portal employs an electronic version of the Frailty Assessment for Care planning Tool (FACT).^18^ Through a combination of patient assessment and caregiver report, the FACT, a structured methodology of the Clinical Frailty Scale,^19,20^ measures the degree of frailty and its drivers (cognition, mobility, function and social circumstances). Clinicians will log into the secure web-based portal and are prompted though a short series of questions, that when combined with collateral responses (ideally from a caregiver), identifies a frailty score.^19,20^ The clinician asks the promoted questions and enters the gathered information into the portal. Collateral reports from caregivers are paper based and used to inform the physicians assessment.^20^ The short set of questions is ideally completed in advance (eg, in the waiting room or while other routine care is being provided). Based on the resulting frailty score, the Frailty Portal provides practical visit goals tailored to the identified frailty level, with links to relevant resources for patients, caregivers and providers. The development of the Frailty Portal was a collaborative effort between Primary Health Care and Department of Family Practice, NSHA, and the Palliative and Therapeutic Harmonization (PATH) program.^15^ Figure shows the steps involved in the use of the Frailty Portal.

**Figure:**
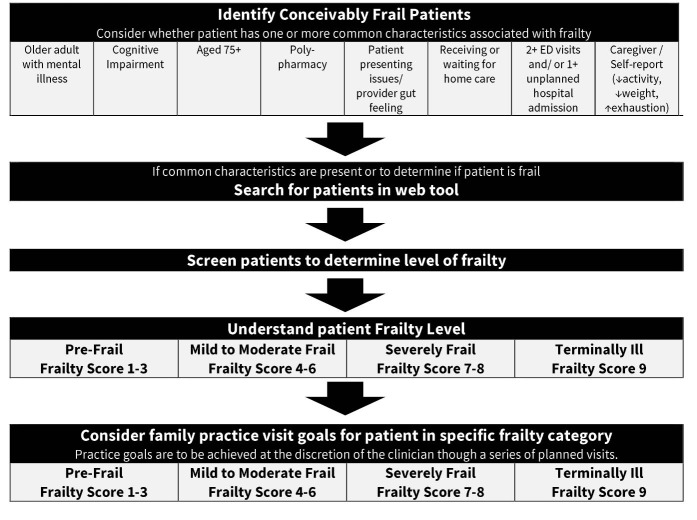
Application of the Frailty Portal in Primary Healthcare (PHC).

Based on the resulting frailty score, the Frailty Portal provides practical visit goals tailored to the identified frailty level, with links to relevant resources for patients, caregivers and providers. The identification of levels of frailty allows patients, caregivers and providers to have a baseline understanding and common language for frailty. Each level of frailty connects to suggested evidence-informed care goals, which the healthcare team implemented collaboratively with patients and caregivers, related to physical activity, healthy diet, and social connection.^1^ The intention is to help frail persons age successfully and maintain independent function as long as possible.^1^ Table 1 shows the levels of frailty.

**Table 1:** Levels of Frailty^17,19^

**Frailty Level**	**Current Ability Level, Symptoms**
**Thriving**	• Fit, exercises regularly • In charge of organizing social events • Still working at a job or actively involved with a skill-based hobby • Impresses others with memory and thinking
**Normal Aging**	• Active, exercises occasionally • Socializes weekly (accompanied by a caregiver as needed) • Completes daily tasks independently, but finds some things challenging • You or your family member is worried about memory
**Vulnerable**	• Starting to slow down and often tired during the day • Socializes less than weekly or a caregiver may not be available to help • Not dependent on others, but symptoms often limit activities • Minor challenges with memory and thinking (not dementia)
**Mild**	• Walking slower and regularly uses (or should use) a cane or walker • Rarely socializes • Needs help with daily tasks and chores (like housework, banking, taking medications) • Vague or incorrect recall of current events
**Moderate**	• Needs help of another person when using stairs, walking or on uneven ground, getting in and out of the bath or has fallen more than once in the past 6 months
**Severe**	• Always needs someone’s help when walking or unable to move self in manual wheelchair • Housebound and isolated • Caregivers may be extremely stressed or there are no available caregivers to meet the person’s care needs • Needs hands on helping with bathing, toileting and dressing • Severe stage dementia; unable to name loved ones
**Very Severe**	• Unable to leave one’s bed, with or without help • Unable to participate in any social exchanges, even when visited • Dependent on others for all aspects of daily life • Very severe dementia, with limited language skills and few spoken words
**Terminal**	• Life expectancy is less than 6 months

Associated with the levels of frailty, visit goals are outlined in the portal as a source of reference for the primary care provider (PCP). Table 2 provides an example of care goals for a patient who has been identified as “vulnerable” through screening. Local community resources are also connected to each goal. Visit goals were developed though peer reviewed evidence and research and consultation with PCPs.

**Table 2:** Example Care Planning Goals

**Frailty Level: Vulnerable**
Goals: • Encourage patient to learn more about personal directives • Discuss regular physical activity • Review medications • Ask about concerns with living and social situations • Discuss and develop crisis planning card • Consider regular reassessment of cognition

#### Study Design and Methodology

This implementation feasibility study used a convergent mixed method design where quantitative and qualitative data will be collected concurrently over a nine month period, then analyzed to understand the feasibility and scalability of the initiative.^18-21^ Using CFIR, we will identify what aspects of the Frailty Portal require adaptation to meet the needs of providers and patients/caregivers and still remain effective. Implementation science is the scientific study of methods to promote the systematic uptake of research findings into routine practice.^18^ Using a framework to guide implementation of research findings is strongly advocated as it allows assessment of factors at multiple levels that may hinder or support the implementation and sustainability of the intervention.^22,23^ CFIR consolidates multiple constructs under five major domains found to influence the successful implementation of innovative programs^18^: (*i*) intervention characteristics, (*ii*) outer setting, (*iii*) inner setting, (*iv*) characteristics of individuals, and (*v*) process.

#### Roles of Participants in the Study

Participating physicians/NPs will clarify the benefits and barriers to integrating the frailty portal into their practice; their understanding of frailty; opinions on whether the portal improves their ability to care for frail older adults; the amount of work involved in using the portal; and if they believe the portal aligns with provincial and professional standards of care. Understanding the point of view of patients and their caregivers will shed light on whether they feel they are more knowledgeable and better informed to make decisions about their health, frailty, and healthcare after having participated in a frailty portal screening, and a discussion about frailty and the associated care goals with their PCP. Input from key stakeholders will provide information about priorities, resources, policies and additional CIFR identified constructs that could potentially influence effective implementation and uptake.

### Study Procedure

The study procedure includes the following steps:

1. Support from the integrated research team: Team members include researchers, geriatricians, family practice physicians, healthcare administrators, decision-makers and on the ground support. Activities include on-going engagement, education and support. Participating providers will attend one full day face-to-face education workshop to receive more in-depth information about identification and the care of the frail and ‘hands-on’ learning of the web-based tools. Workshop sessions will be offered by research team members and PHC information technology leads with support from opinion leaders, community champions and integrated community support service members (eg, home care, family and caregiver supports). On-going support and feedback will be provided by team members, including individual visits as needed. Posters to raise frailty screening awareness among patients and providers will be posted in each participating practice.

2. Portal web-based tools: The portal includes tools for providers to aid the identification, and frailty-level appropriate response to potentially frail patients; downloadable information for patients and caregivers including available community resources; and education materials. In brief, providers are asked to review their practice population and identify potentially frail patients through the use of predetermined cues that may suggest a patient is experiencing frailty. If possible frailty is indicated, they begin the screening process using an electronic version of the FACT^14,16^ to determine the patient’s level of frailty. Input from the patient/caregiver dyad is required at this stage to help inform the final score. Providers then review evidence-based PHC visit goals tailored to match the assigned level of frailty.

3. Integrated community supports: The portal includes a toolkit of available integrated community supports and resources plus referral forms to these programs.

### Data Collection

To accommodate the short time frame of this implementation feasibility study, multiple data collection strategies will be employed concurrently over the first 9 months of the study period.

(*a*) Quantitative count and web-tool audit data: To access provider adherence to implementation activities, counts will be taken to determine the proportion of those taking part in each educational sessions and monthly follow-up opportunities. Similarly the web-tool can provide an audit of provider logins (number, duration), number of patients screened and identified (both partial and completed) and care plan activity.

(*b*) Surveys: Prior to the education workshop, providers will complete a pre-survey to assess their pre-awareness and knowledge of frailty, confidence (self-efficacy competencies), coordination perception and satisfaction with their provision of care for persons experiencing frailty. A similar post survey will be completed at eight months. The pre-survey package will also include a survey to collect provider characteristics (eg, demographics, computer skills), practice setting data (‘inner setting’ eg, team-based, remuneration type, practice population) and information about the care they typically provide to their patients experiencing frailty. During the post period, providers will be asked to complete two additional surveys. The System Usability Scale^21^ to assess provider experience with the web-based tool and a ‘Post Evaluation’ survey where questions are guided by CFIR domains and constructs to assess additional intervention characteristics, inner and outer setting domain constructs such as readiness for implementation and supports for change as well as a reflection and evaluation of the process.

(*c*) Qualitative Information: (1) Providers: Each month providers will be asked to answer short questions about their web-tool use, deviations in adherence, concerns and suggestions. At the end of the study period (9 months), semi-structured interviews will be conducted using questions based on the CFIR to explore the implementation feasibility of the initiative, time required, barriers and facilitators to integrating the portal into their practice and the initiative’s impact on integrated care assess and co-ordination. (2) Patient/provider dyads: Invitations to participate in a short survey will be mailed to patients who were screened, and include a letter that can be passed on to the caregiver. The package will be prepared by the research team but labelled and mailed by the physician (and /or a member of their team). Those who consent to be contacted by the research team will be asked by telephone interview a short set of questions to assess their awareness of frailty and prognosis, care expectations, self-efficacy, perception of care coordination and satisfaction. (3) Key stakeholders: Semi-structured interviews will be conducted with eight to ten key stakeholders to collect information about their priorities, resources, policies and additional CFIR identified constructs potentially influencing the effective implementation and uptake of the initiative. Interviews will be recorded and transcribed (Table 3).

**Table 3:** Key Questions and Data Collection

**Key Questions**
Objective 1. To identify factors influencing the implementation process, feasibility and acceptability of the web-based ‘Frailty Portal’
i. To what extent did providers adhere to the activities of the implementation plan? • Attendance at educational sessions • Practice facilitation uptake • Web-based tool use (patients identified, screened, visit goals reviewed, support referrals) • Modifications made ii. Did providers find the educational workshop useful? • Did the activities increase knowledge of frailty, confidence to provide care? iii. How easy are the web-based tools to use? (access, time, screens) • What are the barriers/facilitators in using the tools? iv. To what extent do providers feel the initiative will aid their provision of appropriate care to the frail?
Objective 2. To begin the examination of the effectiveness of the ‘Frailty Portal’ using targeted preliminary provider, patient, caregiver, and health system outcomes
i. Has provider awareness of frailty and the pre-frail increased? ii. Has provider confidence and satisfaction in providing care for their frail patients increased? iii. Do providers feel the initiative helped them provide better care to their frail patients? iv. Has the initiative resulted in increased FP/PHC access for patients identified as frail? v. Has access to coordinated care and required integrated community supports improved for providers? Patients? Caregivers? vi. Are frail patients and their caregivers more confident in the care they receive? v. Is the initiative associated with reduced unnecessary emergency visits or hospitalizations among identified frail patients?
Objective 3: To identify the core components required to successfully scale up the initiative to a broader community of PHC providers.
i. What intervention, individual (provider, patient, caregiver), setting (inner, outer) and process level factors influence the successful implementation of the initiative? ii. What are the essential components for future scaling up of the ‘Frailty Portal’ initiative?

Abbreviations: PHC, Primary healthcare; FP, family physician.

### Data Analysis Plan

Descriptive statistics will be provided for all quantitative data. Pre-post differences (provider surveys) will be assessed using chi-square analyses and/or appropriate comparison tests such as Wilcoxon signed-rank tests and student *t* tests. Inner and outer setting information will be synthesized to provide a contextual description of PHC practice and the local PHC community to identify factors that facilitate or impede implementation. Qualitative data will be transcribed verbatim and analyzed using framework methodology based on CFIR constructs, and triangulated to begin to identify how participants describe the various aspects of implementation feasibility and perceived impact.^24^ Together this will be used to inform a more robust interpretation of the data to produce a more comprehensive understanding of the Frailty Portal’s feasibility, impact, and identification of core components required for successful scale-up. Qualitative interviews with key stakeholders will be digitally recorded using audio recorders and will occur in person, or (if needed) over the phone. They will be guided by a semi-structured interview guide and tailored to the role of the participant in the project (eg, stakeholder, PCP).

## Discussion

The Frailty Portal is the first web-based tool of its kind in Canada and has garnered interest from other jurisdictions across the country. The goal of the Frailty Strategy of which the ‘Frailty Portal’ is a major part, is to improve access to and the coordination of streamlined services for persons experiencing frailty (including families and caregivers) and to increase family physician and other PHC providers’ ability to care for patients in the context of their frailty.^17,19^ Our goal is to optimize care in the community by helping community PHC providers gain knowledge about frailty, improve the identification of frailty through the use of screening tools, use frailty level specific care goals, and make use of appropriate community supports.^2-4,8,9,11^ This project aims to empower, engage and support patients and their families/caregivers. Improvements in family and caregivers awareness of the meaning of frailty prognosis, opportunity for shared decision-making, and navigation of community supports is expected.

The Frailty Portal is the first step to a community-based model of frailty care. This proposed research will inform our understanding of the prevalence of frailty within our community and provide valuable information about how providers recognize and utilize knowledge of frailty to improve patient and caregiver experience. The project will provide estimates of the time and support required to screen and identify frail patients, develops care goals/crisis management plans, promote informed decision-making. The information gathered will also inform practice structure and clarify what needs to be done to promote effective implementation. The longer term impact is to improve and streamline the care that frail persons receive. In doing so, a reduction in service duplication is expected, as is improved continuity and coordination of care, reduced wait time for provider/community services, greater confidence in the care patients receive by healthcare providers, and improved ability for family/caregiver to understand care issues.

## Ethical issues

Ethical approval was received from the NSHA Ethics Board.

## Competing interests

Authors declare that they have no competing interests.

## Funding

Funding for this project was provided by Technology Evaluation in the Elderly (TVN) TVN File #: FRA2015-B-17.

## Authors’ contributions

Conception and design: BL, TS, and SW; Drafting of the manuscript: SW, TS, BL, and GW; Critical revision of the manuscript for important intellectual content: LM, RG, and PM; Obtaining funding: BL, TS, PM, LGB, FB, RG, and GW; Administrative, technical, or material support: SW.

## Authors’ affiliations

^1^Building Research for Integrated Primary Healthcare (BRIC NS), Nova Scotia Primary & Integrated Health Care Innovations Network, Halifax, NS, Canada. ^2^Primary Care Research Group, Dalhousie Family Medicine, Halifax, NS, Canada. ^3^Dalhousie University, Halifax, NS, Canada. ^4^Primary Health Care, Nova Scotia Health Authority, Halifax, NS, Canada. ^5^School of Occupational Therapy, Dalhousie University, Halifax, NS, Canada. ^6^Continuing Care, Nova Scotia Health Authority, Halifax, NS, Canada. ^7^Healthy Populations Institute, Halifax, NS, Canada. ^8^Palliative and Therapeutic Harmonization (PATH) Program, Halifax, NS, Canada. ^9^Department of Family Practice, Nova Scotia Health Authority, Halifax, NS, Canada.

## Endnotes


[1] Prior to April 1, 2015, known as Primary Health Care and the District Department of Family Practice, Capital District Health Authority.

